# Gene expression profiling of peripheral blood cells: new insights into Ewing sarcoma biology and clinical applications

**DOI:** 10.1007/s12032-014-0109-2

**Published:** 2014-07-10

**Authors:** Joanna Przybyl, Katarzyna Kozak, Hanna Kosela, Slawomir Falkowski, Tomasz Switaj, Iwona Lugowska, Anna Szumera-Cieckiewicz, Konrad Ptaszynski, Beata Grygalewicz, Magdalena Chechlinska, Barbara Pienkowska-Grela, Maria Debiec-Rychter, Janusz A. Siedlecki, Piotr Rutkowski

**Affiliations:** 1 Department of Molecular and Translational Oncology, Maria Sklodowska-Curie Memorial Cancer Center and Institute of Oncology, 5 W.K. Roentgen Street, 02-781 Warsaw, Poland; 2 Department of Human Genetics, KU Leuven and University Hospitals Leuven, UZ Herestraat 49, Box 602, 3000 Louvain, Belgium; 3 Postgraduate School of Molecular Medicine, Warsaw Medical University, 61 Zwirki i Wigury Street, 02-091 Warsaw, Poland; 4 Department of Soft Tissue/Bone Sarcoma and Melanoma, Maria Sklodowska-Curie Memorial Cancer Center and Institute of Oncology, 5 W.K. Roentgen Street, 02-781 Warsaw, Poland; 5 Department of Pathology, Maria Sklodowska-Curie Memorial Cancer Center and Institute of Oncology, 5 W.K. Roentgen Street, 02-781 Warsaw, Poland; 6 Cancer Genetics Laboratory, Department of Pathology, Maria Sklodowska-Curie Memorial Cancer Center and Institute of Oncology, 5 W.K. Roentgen Street, 02-781 Warsaw, Poland; 7 Department of Immunology, Maria Sklodowska-Curie Memorial Cancer Center and Institute of Oncology, 5 W.K. Roentgen Street, 02-781 Warsaw, Poland

**Keywords:** Ewing sarcoma (ES), Circulating tumor cells (CTCs), Hematological abnormalities, Monocytosis, Prognosis, *N*-cadherin

## Abstract

Ewing sarcoma (ES) is a group of highly aggressive small round cell tumors of bone or soft tissue with high metastatic potential and low cure rate. ES tumors are associated with a rapid osteolysis and necrosis. The currently accepted clinical prognostic parameters do not accurately predict survival of high-risk patients. Moreover, neither the subtype of *EWS*–*FLI1/ERG* in the tumor, nor the detection of fusion transcripts in the peripheral blood (PB) samples, has prognostic value in ES patients. We evaluated the prevalence of circulating tumor cells (CTCs) in 34 adult ES patients. Since CTCs were confirmed in only small subset of patients, we further explored the expression profiles of PB leukocytes using a panel of genes associated with immune system status and increased tumor invasiveness. Moreover, we analyzed the alterations of the routine blood tests in the examined cohort of patients and correlated our findings with the clinical outcome. A uniform decrease in *ZAP70* expression in PB cells among all ES patients, as compared to healthy individuals, was observed. Monocytosis and the abnormal expression of *CDH2* and *CDT2* genes in the PB cells significantly correlated with poor prognosis in ES patients. Our study supports the previously proposed hypothesis of systemic nature of ES. Based on the PB cell expression profiles, we propose a mechanism by which immune system may be involved in intensification of osteoclastogenesis and disease progression in ES patients. Moreover, we demonstrate the prognostic value of molecular PB testing at the time of routine histopathological diagnosis.

## Introduction

Ewing sarcoma (ES) family of tumors (morphology code: 9260/3 according to the World Health Organization classification of bone tumors) is a group of highly aggressive small round cell tumors of bone and soft tissues, which occur predominantly in adolescents and young adults [1]. ES entity comprises a few types of tumors: Ewing sarcoma of bones, extraskeletal Ewing tumors, primitive neuroectodermal tumors (PNETs), and Askin tumors, which are actually PNETs of the chest wall [1]. Approximately 25 % of patients present with metastatic disease, which is the most prominent adverse prognostic factor [2]. ES metastasizes predominantly via blood, and the most common sites of metastases include lungs, bones, and bone marrow [2]. Roughly half of the patients with localized disease at diagnosis will relapse within 5 years or later after completing the extensive multimodal therapy [3]. Based on the aggressive clinical course and relatively low curative rate, it has been proposed that ES may be a systemic disease characterized by micrometastases at submicroscopic level [4–7].

Manifestation of systemic disorders frequently includes hematologic abnormalities, such as shift in certain blood cell populations and constitutional inflammation. The initial clinical symptoms of ES may include pain, intermittent fever, anemia, increased white blood cell counts, high C-reactive protein concentration, and sedimentation rate, which are the symptoms of systemic inflammatory reactions [1, 8]. ES tumors are also associated with necrosis and rapid, extensive osteolysis [9, 10]. Osteoclast formation in ES is mediated by RANKL-dependent pathway and promoted by the tumor-associated macrophages (TAMs) [8, 9, 11]. TAMs may foster local invasion and are considered as one of the key regulators of cancer-associated inflammation [12]. Macrophage infiltration is an adverse prognostic factor in ES and in many other tumor subtypes including bladder, breast, cervical, and prostate cancers [8]. Also, tumor necrosis facilitates neoplastic progression and has a pro-inflammatory potential [12].

ES tumors are characterized by oncogenic chromosomal translocations detected in 85–95 % of cases. The most common are predominantly balanced t(11;22)(q24;q12) and t(21;22)(q22;q12) translocations, resulting in *EWS*–*FLI1* and *EWS*–*ERG* fusion gene formation, respectively [13, 14]. *EWS* gene (also known as *EWSR1*—Ewing sarcoma breakpoint region 1) encodes a multifunctional protein involved in DNA-dependent regulation of transcription. *FLI1* (Friend leukemia virus integration 1, also known as *EWSR2*) and *ERG* [v-*ets* erythroblastosis virus E26 oncogene homolog (avian)] are closely related members of the erythroblast transformation-specific (ETS) family of transcription factors, containing ETS-type DNA-binding domain [15, 16]. The oncogenic fusion proteins preserve the N-terminal EWS domain and ETS domain of FLI1 or ERG, and function as an aberrant transcriptional activator [14, 17, 18]. In the most prevalent fusion variants, *EWS* exon 7 is fused to *FLI1* exon 6 (type 1), *FLI1* exon 5 (type 2), or *ERG* exon 9 [14, 15]. However, it has been well documented that multiple splice variants of *EWS*–*FLI1* fusion genes may be co-expressed within the same ES tumor [15, 17, 19].

Detection of *EWS* rearrangements by RT-PCR and/or fluorescence in situ hybridization (FISH) in formalin-fixed paraffin-embedded tissue specimens has become a routine practice in molecular diagnosis of ES [20–22]. Nevertheless, it has been agreed that the fusion subtype does not affect prognosis [23–25]. There are several immunohistochemical markers useful in ES diagnosis, but none of them is exclusively ES-specific. CD99 (encoded by *MIC2* gene) is expressed in virtually all cases in a characteristic membranous pattern [26]. Vimentin is also expressed in a large subset of ES tumors [2, 27]. More differentiated ES tumors, especially PNETs, are immunopositive for neural markers such as neuron-specific enolase (NSE) or S-100 protein [2, 28]. Molecular and histological diagnosis of ES is based on tissue specimen obtained with a core or open biopsy, but examination of such material may be hampered by several factors associated with poor representativeness or inadequate fixation method. Biopsy is an invasive procedure that may be accompanied by technical problems due to difficult tumor location or bone sclerosis. Biopsy may also cause discomfort suffered by the patient and subsequent surgical complications. To overcome tumor sampling limitations, sensitive techniques for “liquid biopsy” analysis to monitor cancer genetics in blood have been extensively developed in the recent years [29, 30]. Blood-based molecular biomarkers are likely to be clinically useful, especially for early diagnosis, prognosis, and selection of specific personalized therapy [30]. Several groups have already shown that circulating tumor cells (CTCs) carrying *EWS*–*FLI1/ERG* fusion transcripts may be detected in 6–43 % of the peripheral blood (PB) specimens of ES patients at the time of diagnosis but the prognostic significance of these findings remains disputable [4, 5, 31–35]. It has also been demonstrated that gene expression profiling of peripheral blood mononuclear cells (PBMCs) may identify specific functional abnormalities associated with disease outcome and response to therapy in patients with solid tumors [36–42]. Numerous immunological tumor–host interactions involve certain subsets of PBMCs; thus, their gene expression profiles may provide a comprehensive picture of patient’s immune status.

Since the expression of specific fusion genes lacks prognostic significance in ES, microarray technology has been applied to identify prognostically relevant secondary genetic alterations. As a result, numerous genes and pathways associated with the aggressive course of disease have been recognized, including Wnt-signaling, TP53, PI3 kinase pathways, as well as genes involved in cell adhesion, regulation of transcription, and cell cycle control [43–46]. Specifically, the overexpression of *CDH11* and *MTA1*, and down-regulation of *CDH2* have been observed in ES patients with poor prognosis [43]. Elevated *CCND1* and *STEAP1* expression in tumor and bone marrow specimens has been found to correlate with worse patients’ survival [44]. Overexpression of *CCND1* has also been detected in primary tumors of ES patients at high risk of metastasis development [45]. *CDT2* (also known as *DTL*) overexpression is another recently identified biomarker of adverse prognosis in ES patients, and it was proposed that the ubiquitin ligase inhibitors may serve as therapeutic agents targeting CDT2 activity [46, 47]. The most important functions of the above-mentioned genes are listed in Table 1.

**Table 1:** Selected gene-related functions of adverse prognostic markers identified in ES tumors, according to the NCBI AceView database (http://www.ncbi.nlm.nih.gov/IEB/Research/Acembly/index.html)

No	Gene symbol	Gene name	Function
1	*CCND1*	Cyclin D1	Cyclin-dependent protein kinase regulator activity, protein kinase activity, cell division, regulation of cell cycle
2	*CDH2*	Cadherin 2, type 1, *N*-cadherin (neuronal)	Cell adhesion, calcium-dependent cell–cell adhesion, cell migration
3	*CDH11*	Cadherin 11, type 2, OB-cadherin (osteoblast)	Cell adhesion, skeletal system development
4	*CDT2*	Denticleless homolog (Drosophila)	Ubiquitin–protein ligase activity (protein mono- and polyubiquitination), regulation of cell cycle (G2/M transition DNA damage checkpoint), DNA replication
5	*MTA1*	Metastasis associated 1	Transcription factor activity; regulation of transcription, DNA-dependent
6	*STEAP1*	Six transmembrane epithelial antigen of the prostate 1	Transporter activity, channel activity

Based on the available tumor-derived data, we sought to identify molecular prognostic markers that could be evaluated in the PB specimens, used as so-called liquid biopsies. We aimed to assess whether the presence of *EWS*–*FLI1/ERG* fusion transcripts that characterize CTCs in ES patients correlate with disease outcome. We also studied the expression levels of a panel of genes related to immune status such as *ZAP70*, *NFKB1*, *IL8*, and *IL2RA*, and increased tumor invasiveness such as *CDT2*, *CDH2,* and *MTA1*, in the PB cells of ES patients. Moreover, we analyzed the alterations in routine blood tests of these patients and correlated our findings with the clinical data. Our study provided a new insight into ES biology and identified potential prognostic markers that can be examined in the PB samples drawn from ES patients at the time of diagnosis.

## Materials and methods

### Ethics statement

PB specimens were collected after written informed consent had been obtained, according to the protocol approved by the Bioethical Committee of the Maria Sklodowska-Curie Memorial Cancer Center and Institute of Oncology (MSCMCCIO), Warsaw, Poland.

The archival frozen tumor samples obtained from KU Leuven and University Hospitals, Belgium, originate from patient care and were requalified for research. The Ethical Committee of KU Leuven and University Hospitals, Belgium, approved the study.

### Study population and ES diagnosis

Thirty-four untreated adult patients diagnosed with ES (20 females and 14 males; median age at diagnosis 27 years, range 19–59 years) and 13 healthy individuals (7 females and 6 males, median age 37 years, range 23–68 years) were recruited for this prospective study (Table 2) between July 2008 and October 2011 at the MSCMCCIO.

**Table 2:** Clinical, pathological, and molecular features of patients with ES family of tumors

No	Nested RT-PCR in PB	FISH assay	Sex	Age	Primary site	Size of primary tumor (cm)	M0/M1 status at diagnosis	Last follow-up (months/latest status)
1	Neg	Pos	F	24	Iliac bone	>10	M1	33/DOD
2	Neg	NA	M	19	Tibia	10	M0	62/AWOD
3	Neg	NA	F	20	Paraspinal	NA	M1	15/DOD
4	Neg	Pos	M	59	Scapula	6	M0	61/AWOD
5	Neg	NA	F	29	Femur	NA	M0	53/AWOD
6	Neg	Pos	F	29	Ischium	>10	M0	5/DOD
7	Pos (*EWS*–*ERG*)	Pos	M	42	Iliac bone	>10	M1	9/DOD
8	Neg	Pos	F	20	Femur	14	M1	7/DOD
9	Neg	Pos	F	25	Iliac bone	>10	M1	55/AWD
10	Neg	Pos	M	33	Femur	7	M0	50/AWOD
11	Neg	NA	M	37	Chest wall	15	M0	49/AWOD
12	Neg	Pos	F	40	Tibia	>10	M0	7/DOD
13	Neg	NA	M	24	Chest wall	>10	M1	4/DOD
14	Neg	NA	F	19	Femur	>10	M0	1/AWD
15	Neg	Pos	F	19	Scapula	9.5	M0	44/AWOD
16	Neg	Pos	M	22	Femur	11	M1	6/DOD
17	Neg	NA	F	33	Forearm	7	M0	43/AWOD
18	Neg	NA	M	20	Humerus	NA	NA	NA/AWD
19	Neg	Pos	M	25	Fibula	10	M0	15/DOD
20	Neg	Pos	M	27	Costa	NA	M0	34/DOD
21	Neg	NA	F	37	Paraspinal	12	M0	14/DOD
22	Neg	NA	F	30	Thigh	8.5	M1	25/DOD
23	Neg	NA	F	30	Femur	NA	M0	37/AWD
24	Neg	NA	F	24	Retroperitoneal space	NA	M0	37/AWD
25	Neg	Pos	F	25	Forearm	9.5	M0	33/AWOD
26	Neg	NA	F	34	Humerus	NA	M0	34/AWD
27	Neg	NA	F	27	Pelvis	NA	M1	14/DOD
28	Neg	Pos	M	41	Abdominal wall	7	M1	34/AWD
29	Pos (*EWS*–*FLI1* type 2)	Pos	F	23	Pelvis	NA	M1	3/DOD
30	Neg	NA	M	43	Femur	NA	M0	10/DOD
31	Neg	NA	M	20	Ribs	17	M0	28/AWOD
32	Neg	Pos	F	29	Mediastinum	15	M0	111/AWD
33	Pos (*EWS*–*FLI1* type 1)	NA	F	21	Ischium	8	M1	18/DOD
34	Neg	NA	M	35	Femur	13	M0	26/AWOD

*RT-PCR* reverse transcription polymerase chain reaction, *FISH* fluorescent in situ hybridization, *M0* no evidence of metastasis, *M1* evidence of metastasis, *Pos* positive, *Neg* negative, *NA* not available, *DOD* dead of disease, *AWD* alive with disease, *AWOD* alive without disease

The diagnosis of ES was established on FFPE tissue specimens using standard pathologic criteria and supported by immunohistochemistry and FISH analysis in the Department of Pathology, MSCMCCIO. FISH analysis for the detection of *EWS* gene rearrangement using LSI EWSR1 (22q12) dual-color, break-apart rearrangement probe (Abbott Molecular) was performed using standard methods. Tumors with negative or non-informative FISH result were diagnosed as ES based on the combination of morphological and immunohistochemical features, evaluated independently by two pathologists (A.S.C. and K.P.). Differential diagnosis excluded cancer, lymphoma, melanoma, rhabdomyosarcoma, synovial sarcoma, and other sarcoma subtypes in these cases.

### Peripheral blood (PB) specimens’ preparation and extraction of total RNA

Approximately 10 ml of PB was drawn into EDTA tubes (250 μl, 0.5 M, pH = 8) before the diagnostic biopsy and the beginning of a multimodal chemotherapy, radiotherapy, and surgery (when possible) according to the established treatment protocols [48]. Each PB specimen was divided into two portions and centrifuged (3000 rpm, 10 min, at room temperature) in order to separate blood cells from plasma. The bottom fraction containing red blood cells and the buffy coat containing leukocytes, platelets, and circulating tumor cells (CTCs) were used to extract total RNA with TRI Reagent BD (Sigma) and subsequent DNase treatment using RNease-free DNase set (Qiagen, Valencia, CA, USA), according to the manufacturers’ protocols. RNA quantity and quality were evaluated using NanoDrop 2000 Spectrophotometer (Thermo Scientific, Waltham, MA, USA) and FlashGel system (Lonza, Basel, Switzerland).

### Hematological analysis

Standard hematological parameters were evaluated by laboratory blood tests in ES patients and healthy individuals at the time of diagnosis, as a routine admission procedure at the MSCMCCIO. PB specimens for routine blood tests and for this study were drawn at the same time. We evaluated the following parameters: hemoglobin level (HGB), platelet count (PLT), white blood cell count (WBC), neutrocyte count (NE), lymphocyte count (LY), and monocyte count (MO).

### Tumor specimens and ES cell line

Archival frozen tumor specimens and RD-ES cell line served as positive controls for nested RT-PCR experiments. Frozen tumor samples from 5 untreated ES patients were obtained from KU Leuven and University Hospitals, Belgium (4 primary tumors and 1 metastatic tumor; 4 males and 1 female; median age 25 years, range 4–43 years). Total RNA from the frozen tumor specimens was extracted using miRNeasy kit (Qiagen) including DNase treatment using RNease-free DNase set (Qiagen) according to the manufacturer’s protocol. RNA quantity was determined using NanoDrop 2000 Spectrophotometer (Thermo Scientific), and the quality was evaluated using Bio-Rad Experion RNA StdSens Analysis system (Bio-Rad, Hercules, CA, USA). RD-ES cell line carrying *EWS*–*FLI1* type 2 translocation was purchased from CLS Cell Lines Service GmbH. Total RNA was extracted from RD-ES cells using RNeasy kit (Qiagen) according to the manufacturer’s protocol. RNA quantity and quality were evaluated using NanoDrop 2000 Spectrophotometer (Thermo Scientific) and FlashGel system (Lonza), respectively.

### Isolation and culture of PBMCs

Peripheral blood mononuclear cells (PBMCs) specimens were obtained from the Department of Immunology, MSCMCCIO. PBMCs of 3 healthy donors were obtained by standard Ficoll-Paque (Pharmacia/Pfizer, New York City, NY, USA) gradient centrifugation. Samples of non-cultured cells were taken, and the remaining cells were cultured in a standard RPMI-1640 medium with l-glutamine (Gibco/Thermo Scientific, Waltham, MA, USA), supplemented with 10 % heat-inactivated FCS (Gibco) and gentamycin (Sigma, 50 μg/ml), for 4 days in the presence of phytohemagglutinin (PHA, Wellcome/GlaxoSmithKline, Brentford, England; 1 μg/ml). Cells were centrifuged, washed twice in PBS, and the cell pellet was kept frozen at −70 °C until RNA was isolated. Total RNA was isolated from PBMCs with the use of RNeasy kit (Qiagen). The quantity and quality of the RNA were examined using NanoDrop 2000 Spectrophotometer (Thermo Scientific) and by denaturing gel electrophoresis with ethidium bromide.

### Nested reverse transcription PCR (nested RT-PCR)

Nested RT-PCR was performed to detect *EWS*–*FLI1/ERG* fusion transcripts in CTCs and tumor specimens, according to the modified protocol described by Peter et al. [31], which allows detection of 1 tumor cell per million of mononuclear blood cells. One microgram of total RNA was reverse-transcribed with oligo(dT)_12–18_ primers and random hexamers using SuperScript II Reverse Transcriptase (Invitrogen/Thermo Scientific, Waltham, MA, USA). cDNA quality was assessed by PCR using GAPDH primers (Table 3).

**Table 3:** Primers used for RT-PCR, nested RT-PCR, and direct sequencing

Designation	Sequence	Direction	NCBI reference sequence
GAPDH1	5′ GGTCGGAGTCAACGGATTTG 3′	Forward	NM_002046.4
GAPDH2	5′ ATGAGCCCCAGCCTTCTCCAT 3′	Reverse	NM_002046.4
22.8	5′ CCCACTAGTTACCCACCCCAAA 3′	Forward	NM_013986.3
22.3	5′ TCCTACAGCCAAGCTCCAAGTC 3′	Forward	NM_013986.3
FLI11	5′ AGGGTTGGCTAGGCGACTGCT 3′	Reverse	NM_002017.4
FLI3	5′ GTCGGGCCCAGGATCTGATAC 3′	Reverse	NM_002017.4
ERG11	5′ TGTTGGGTTTGCTCTTCCGCTC 3′	Reverse	NM_182918.3
ERG3	5′ ACTCCCCGTTGGTGCCTTCC 3′	Reverse	NM_182918.3

*EWS*–*FLI1* and *EWS*–*ERG* fusion junctions were detected using AmpliTaq Gold DNA Polymerase (Invitrogen). The first round of amplification was performed with 2.5 μl of cDNA using primers appropriate for *EWS* and *FLI1/ERG* genes (22.8 and FLI11/ERG11, respectively), and the second round of amplification was performed using one-fifth of the first round PCR product, with internal primers appropriate for *EWS* and *FLI1/ERG* genes (22.3 and FLI3/ERG3, respectively) (Table 3). The first and second round of amplifications were performed in 25 μl volumes at 94 °C for 30 s, 68 °C for 60 s, and 72 °C for 60 s for 15 and 35 cycles, respectively, and the final extension was performed for 10 min in both reactions. cDNA from RD-ES cell line and frozen tumor specimens were used as positive controls. Appropriate negative controls were also included in every step of the procedure. PCR products were visualized by electrophoresis in 2 % TBE agarose gels stained with ethidium bromide, purified using ExoSAP-IT, PCR Product Clean-Up (Affymetrix, Santa Clara, CA, USA) and prepared for direct sequencing using BigDye^®^ Terminator version 3.1 Cycle Sequencing Kit (Life Technologies) with subsequent precipitation using ExTerminator kit (A&A Biotechnology, Gdynia, Poland). Samples were analyzed using the 3130×L Genetic Analyzer (Life Technologies).

### Quantitative reverse transcription PCR (qRT-PCR)

Five hundred nanogram of total RNA was reverse-transcribed with oligo(dT)_12–18_ primers and random hexamers using High-Capacity cDNA Reverse Transcription kit (Life Technologies). cDNA quality was assessed by PCR using GAPDH primers (Table 3). All reactions were performed in duplicate or triplicate using the TaqMan Gene Expression Assays (Life Technologies) (Table 4) in the 7500 Fast Real-Time PCR System (Life Technologies), according to the manufacturer’s recommendations. Experimental data were analyzed using threshold-cycle (Ct) values generated in the SDS 2.1 software (Life Technologies). Mean expression level of two reference genes *PSMC4* and *EIF2B1* served as endogenous control. Endogenous controls were selected from a set of 4 candidate genes (*EIF2B1, MRPL19*, *POP4*, and *PSMC4*) based on the most stable expression across different sample types (blood, tumor tissue, and cell line), which was calculated using geNorm [49] and NormFinder [50] algorithms. Negative controls were also included in every step of the procedure. Total RNA from RD-ES cell line served as an inter-plate calibrator.

**Table 4:** TaqMan assays (Applied Biosystems) used in qRT-PCR experiments

Gene symbol	Assay category	Assay ID
*PSMC4*	Control assay	Hs00197826_m1
*EIF2B1*	Control assay	Hs00426752_m1
*CCND1*	Gene expression assay	Hs00765553_m1
*CDH1*	Gene expression assay	Hs01023894_m1
*CDH2*	Gene expression assay	Hs00983056_m1
*CDH11*	Gene expression assay	Hs00901475_m1
*CDT2*	Gene expression assay	Hs00978565_m1
*MTA1*	Gene expression assay	Hs00950776_m1
*STEAP1*	Gene expression assay	Hs00185180_m1
*IL8*	Gene expression assay	Hs00174103_m1
*IL2RA*	Gene expression assay	Hs00907779_m1
*NFKB1*	Gene expression assay	Hs00765730_m1
*ZAP70*	Gene expression assay	Hs00896347_m1

### Data analysis

Chromas Lite 2.01 software (Technelysium Pty, Ltd, South Brisbane, Australia) and BLAST software were used for the analysis of sequencing data (http://blast.ncbi.nlm.nih.gov/Blast.cgi).

Relative gene expression levels were calculated using 2^−ΔΔC*t*^ method [51]. Statistical analysis was performed using GraphPad QuickCalcs (Grubbs’ test to detect outliers; *t* test to compare two means between groups; chi-square test for contingency tables analysis) (GraphPad Software, Inc., La Jolla, CA, USA).

For the survival analysis, the Kaplan–Meier estimator was used with the log-rank tests for bivariate comparisons using STATISTICA 7.0 software (Statsoft, Tulsa, OK, USA). Overall survival (OS) time for the assessment of prognostic value of clinical and molecular parameters was calculated from the date of start of therapy to the date of the most recent follow-up (censored data) or death. The differences were considered statistically significant if the *p* values were below 0.05.

## Results

### Detection of *EWS*–*FLI1/ERG* fusion transcripts

Total RNA and cDNA were obtained from PB specimens of all 34 patients. CTCs carrying typical *EWS*–*FLI1* and *EWS*–*ERG* transcripts were detected in 19 % (*n* = 3) of patients with positive FISH result in FFPE tumor specimen. Typical type 1 or 2 *EWS*–*FLI1* transcripts were detected in all five frozen tumor specimens and RD-ES cell line used as controls. Specificity of nested RT-PCR blood tests for both *EWS*–*FLI1* and *EWS*–*ERG* detection was 100 % (95 % CI 73.35 to 100.0 %).

**Fig. 1:**
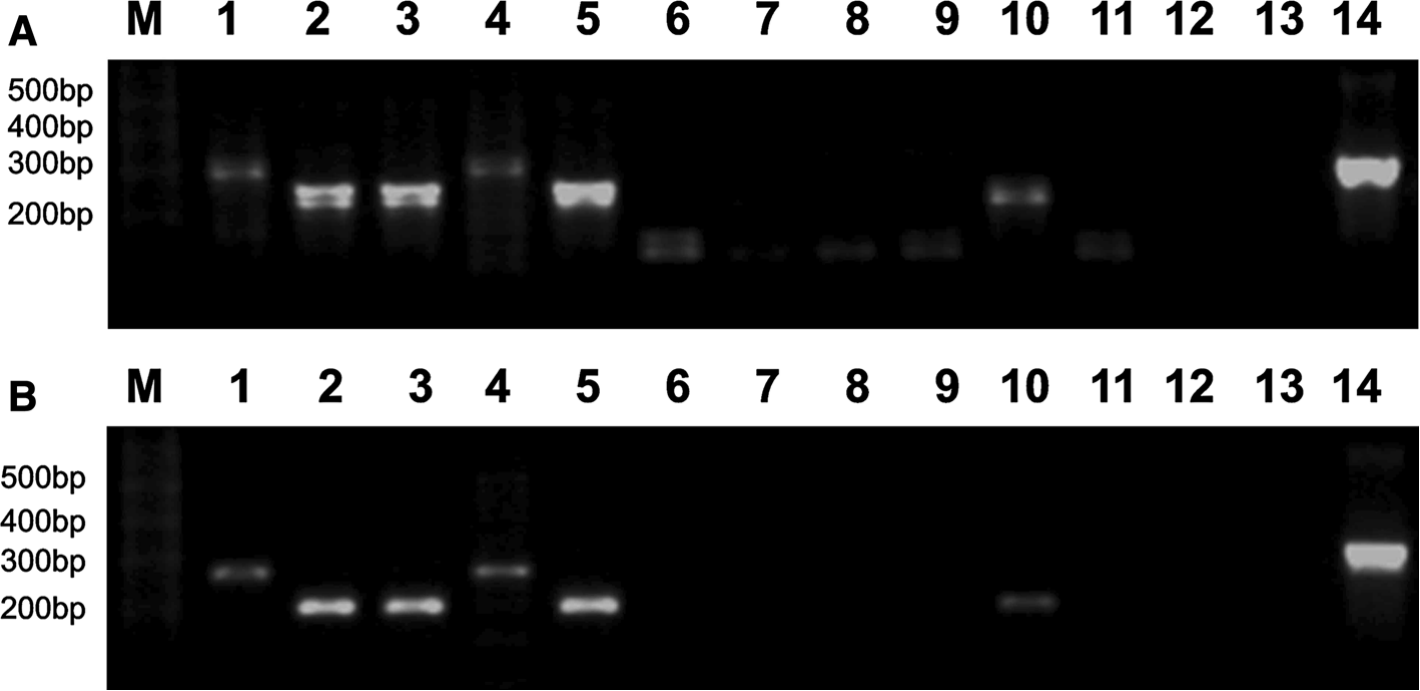
Nested RT-PCR products obtained from the control frozen tumor tissues and PB specimens of ES patients visualized by electrophoresis in 2 % agarose gel stained with ethidium bromide. **a** The second round of amplification was performed using one-fifth of the first round PCR product. **b** The second round of amplification was performed using one-fifth of the 10× dilution of the first round PCR product. M: 1-kb Plus DNA ladder (Invitrogen). *Lanes 1–5* PCR products from frozen tumor tissues; *lanes 6–11* PCR products from selected PB specimens from ES patients; *lanes 12, 13* PCR negative controls for two rounds of amplification; *lane 14* positive control, *EWS*–*FLI1* type 2 fusion (RD-ES cell line). *Lanes 2, 3, 5,* and *10* *EWS*–*FLI1* type 1 fusions, *lanes 1* and *4* *EWS*–*FLI1* type 2 fusions

In 76 % (*n* = 26) of ES patients, the unexpected, shorter *EWS*–*FLI1* transcripts were detected; however, these transcripts were not detectable when 10× dilution of the first round PCR product was used for nested amplification (Fig. 1). Nevertheless, the sequence analysis revealed that these unusual rearrangements cause a shift of the reading frame, resulting in the introduction of premature STOP codons. The predicted truncated chimeric proteins do not preserve any functional domains of FLI1 protein, and therefore, most likely are not oncogenic.

### Hematological abnormalities in ES patients

Results of the laboratory blood tests were available for 33 ES patients and 10 healthy individuals. One or more parameters outside the reference range were found in 85 % (*n* = 29) of ES patients. The most frequent blood test alterations in ES patients were decreased HGB level (67 %; *n* = 22), monocytosis (36 %; *n* = 12), lymphocytopenia (36 %; *n* = 12), thrombocytosis (27 %; *n* = 9) and neutrophilia (27 %; *n* = 9), and increased WBC count (21 %; *n* = 7). Anemia, as defined by WHO criteria [52], was observed in 52 % (*n* = 17) of ES patients at the enrollment. Hematological data for ES patients and healthy individuals are summarized in Tables 5 and 6.

**Table 5:** Results of standard hematological tests in ES patients

	WBC (× 10^9^/l)	HGB (g/dl)	PLT (× 10^9^/l)	NE (× 10^9^/l)	LY (× 10^9^/l)	MO (× 10^9^/l)
Mean ± SD	8.07 ± 3.76	11.98 ± 1.66	334.7 ± 103.64	5.4 ± 3.01	1.8 ± 0.82	0.83 ± 0.51
*Above the reference upper limit*						
n	7	0	9	9	2	12
%	21	0	27	27	6	36
*Below the reference lower limit*						
n	3	22	1	6	12	1
%	9	67	3	18	36	3

*WBC* white blood cell count, *HGB* hemoglobin level, *PLT* platelet count, *NE* neutrocyte count, *LY* lymphocyte count, *MO* monocyte count, *l* liter, *g/dl* gram per deciliter, *SD* standard deviation

**Table 6:** Results of standard hematological tests in healthy individuals

	WBC (×10^9^/l)	HGB (g/dl)	PLT (×10^9^/l)	NE (×10^9^/l)	LY (×10^9^/l)	MO (×10^9^/l)
Mean ± SD	9.32 ± 5.67	13.66 ± 1.37	252.10 ± 83.17	6.36 ± 4.79	2.05 ± 0.77	0.64 ± 0.41
*Above the reference upper limit*						
n	1	0	1	2	2	1
%	10	0	10	20	20	10
*Below the reference lower limit*						
n	0	3	0	0	2	0
%	0	30	0	0	20	0

*WBC* white blood cell count, *HGB* hemoglobin level, *PLT* platelet count, *NE* neutrocyte count, *LY* lymphocyte count, *MO* monocyte count, *SD* standard deviation

### Gene expression results

**Fig. 2:**
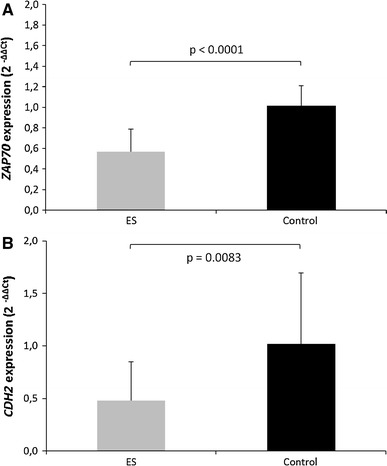
Analyses of the relative gene expression level of **a** *ZAP70* and **b** *CDH2* genes performed by qRT-PCR, using RNA samples from PB cells of ES patients and healthy individuals. The error bars represent standard deviation from the mean 2^−ΔΔC*t*^

cDNA from 23 ES patients, 9 healthy individuals, and RD-ES cell line was available for quantitative gene expression experiments. We observed significant down-regulation of *ZAP70* expression in all ES patients as compared to healthy controls (*p* < 0.0001) (Fig. 2a). Also, *CDH2* was significantly down-regulated in the majority of ES patients (*p* = 0.0083) (Fig. 2b), with more than twofold decrease in the relative *CDH2* expression level in PB cells observed in 14 ES patients. Moreover, *CDT2* and *IL8* were significantly overexpressed, each in 4 ES patients (*p* < 0.0001 and *p* = 0.0047, respectively). The upper limit of normal *CDT2* and *IL8* expression level was defined as mean expression level +2 standard deviations (SD) in healthy controls.

Expression of *STEAP1* and *CDH11* could not be detected in any of the PB specimens. No significant differences between ES and control PB specimens were observed regarding *CCND1*, *CDH1*, *MTA1, IL2RA,* and *NFKB1* expression.

Mitogen-activated lymphocytes were characterized by significant down-regulation of *ZAP70* expression (*p* = 0.017), and overexpression of *CDT2* (*p* = 0.008), as compared to the unstimulated PBMCs.

### Correlation of hematological and genetic findings in PB with the clinical outcome

The median follow-up for all 34 ES patients involved in this study was 28 months (range 1–111 months). At the time of last follow-up, 30 patients completed the treatment procedure, two patients continued therapies in other centers, in two cases the therapy was interrupted because of patient’s death. During the follow-up period, 16 patients died from disease and another 3 patients had documented progression of the disease.

All three patients with confirmed CTCs had distant metastases at the time of diagnosis and died from ES during follow-up.

**Fig. 3:**
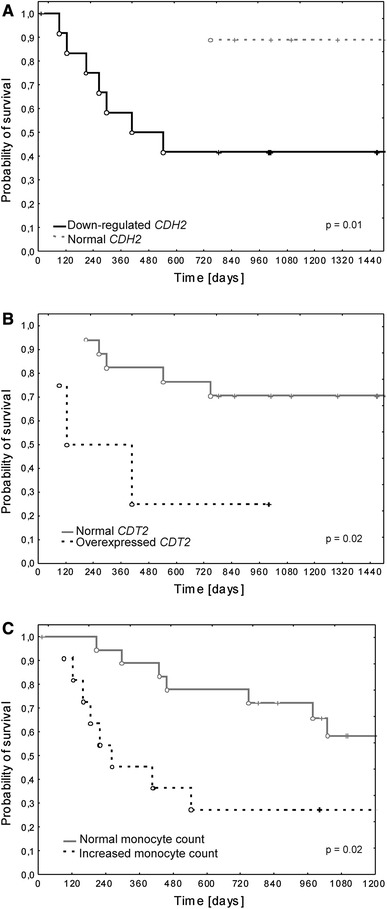
Kaplan–Meier analysis of overall survival (OS) according to **a** at least twofold decrease in *CDH2* expression level and **b** *CDT2* overexpression and **c** monocytosis in PB specimens

The Kaplan–Meier analysis demonstrated significantly worse OS in patients with at least twofold *CDH2* down-regulation and *CDT2* overexpression in PB cells at the time of enrollment (*p* = 0.01 and *p* = 0.02, respectively) (Figs. 3a, b). Also, monocytosis correlated with significantly shorter OS in ES patients (*p* = 0.02) (Fig. 3C). *IL8* overexpression did not affect the probability of survival (*p* = 0.3).

## Discussion

We demonstrated the presence of CTCs carrying oncogenic *EWS*–*FLI1/ERG* fusions in only 19 % (*n* = 3) of ES patients with confirmed *EWS/FLI1* rearrangements in tumor specimen. Roughly comparable chimeric transcript detection rates in PB specimens of ES patients at diagnosis have been also reported in previously published series [31, 34, 35]. Detection of CTCs in only small subset of patients prompted us to investigate hematological profiles and expression levels of genes related to immune status and increased tumor invasiveness in PB specimens of ES patients. We selected *CCND1*, *MTA1*, *STEAP1*, *CDH2*, *CDH11,* and *CDT2* genes that are known to be involved in metastatic spread in several sarcoma subtypes, and their abnormal expression in tumors correlates specifically with poor prognosis in ES patients [43–46]. We also examined the expression level of *CDH1* gene, encoding E-cadherin, which mediates the formation of chemoresistant spheroids from non-adherent ES cells by the activation of the ERBB4 tyrosine kinase, induction of the PI3K-AKT pathway, and suppression of anoikis [53]. In addition, we chose four genes that would provide an insight into different aspects of immune status of ES patients. *ZAP70* encodes cytoplasmic tyrosine kinase, which plays a central role in the initiation of T-cell response [54]. *NFKB1* codes for a DNA-binding subunit of the NF-kappa-B (NFκB) protein complex transcription regulator, and altered expression of this gene is associated with cancer and many inflammatory diseases [55]. Interleukin 8 (IL-8) is a pro-inflammatory chemokine that promotes angiogenesis, tumor progression, invasion, and metastasis [56]. IL-8 is expressed in monocytes and macrophages, and both macrophage infiltration and high expression level of *IL8* correlate with poor outcome in ES patients [8, 57]. *IL2RA* encodes a soluble subunit of IL-2 receptor expressed on activated lymphocytes [58].

We observed a uniform decrease in *ZAP70* expression among all ES patients compared to healthy individuals. An impaired expression of ZAP70 and/or associated zeta chain protein was previously described in activated circulating T cells, and connected with poor prognosis of patients with head and neck, lung, laryngeal squamous cell carcinoma, and melanoma [59–62]. ZAP70 is rapidly degraded in antigen-activated T lymphocytes, in parallel with zeta chain and T-cell receptors (TCRs) [63]. Also, in our specimens of normal mitogen-activated T lymphocytes, *ZAP70* was significantly down-regulated as compared to unstimulated PBMCs. Moreover, the lack of *NFKB1* activation in the circulating leukocytes of the examined ES patients argues for the inactivation of ZAP70 signaling pathway. We suggest that decreased expression of *ZAP70* may reflect a sustained activation of circulating T cells. This assumption goes in line with the previous evidence of circulating tumor-reactive T lymphocytes in ES patients [64]. Moreover, elevated *CDT2* expression level in PB in a subset of ES patients may be linked with the presence of activated circulating T lymphocytes. Our results in normal PBMCs, just like the data retrieved from the Genevestigator database (www.genevestigator.com/gv/biomed.jsp) [65], showed *CDT2* overexpression following stimulation. We propose that there may be an apparent interplay between activated T lymphocytes and ES cells, since both can trigger RANKL-mediated osteoclastogenesis [9, 66, 67]. Osteoclasts differentiate from the proliferative monocyte fraction of PBMCs and from TAMs [11, 68]. Also, TAMs themselves derive from the circulating monocytes that are selectively attracted to the tumor microenvironment [69]. We speculate that there may be a mechanism linking the aggressive course of ES and T-cell activation, intensified osteoclast formation, bone lysis, and necrosis. In our study, in over one-third of patients, we demonstrated monocytosis, which significantly correlated with worse disease outcome. Moreover, we observed worse OS in patients with a strong decrease in *CDH2* expression in PB cells, and *CDH2* down-regulation is a characteristic feature of osteoclasts and their precursors [70, 71]. There was a significant correlation between monocyte count and *CDH2* expression level in our study (*p* = 0.04), and we noted at least twofold *CDH2* down-regulation in all but one of the patients with monocytosis. Interestingly, monocytosis is frequently induced by necrosis [72], and TAMs tend to accumulate into necrotic regions of tumors [69]. This interpretation further supports the notion that ES is a systemic disease; however, to verify this concept, elaborate functional studies are necessary.

Our study shows that adult ES patients present specific hematological abnormalities. Alterations in routine blood tests have previously been examined in soft tissue sarcoma patients; however, correlations of these parameters with particular histological subtypes have not been examined [73]. In our cohort of ES patients, hematological disturbances were observed in 85 % of cases, which is twice as much than Ruka et al. [73] have shown in other sarcoma subtypes. Most prevalent hematological alterations observed across diverse sarcoma subtypes were neutrophilia, leucocytosis, decreased HGB level, monocytosis, and thrombocytosis, in descending order [73]. In ES patients, frequencies of blood test abnormalities were different, with decreased HGB level being the most frequent, followed by monocytosis, lymphocytopenia, thrombocytosis, neutrophilia, and increased WBC count. These abnormalities did not correlate with clinicopathological features, such as metastatic status or tumor size at diagnosis. Considering the increased monocyte count and the above-described molecular findings, there is a possibility that the proliferative fraction of circulating monocytes plays a significant role in ES biology.

**Fig. 4:**
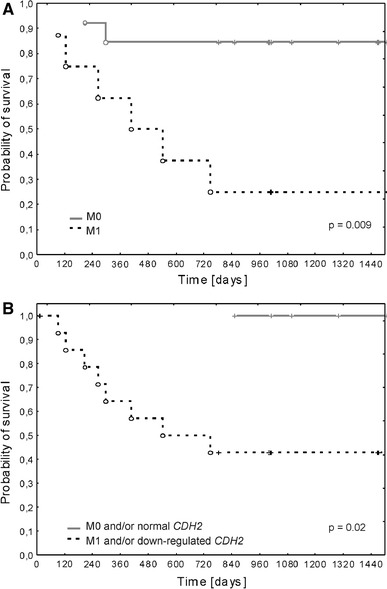
Kaplan–Meier analysis of overall survival (OS) according to **a** M0/M1 status at diagnosis, and **b** M1 at diagnosis combined with at twofold decrease in *CDH2* expression level

The most important clinical implication of our findings is a considerable *CDH2* down-regulation in PB specimens of patients with worse OS. Decrease in *CDH2* expression has previously been correlated with poor prognosis in ES, rhabdomyosarcoma, and Wilms’ tumors [43]. It has also been demonstrated that both *CDH2* and *N*-cadherin down-regulations dramatically change glioma cells migratory behavior and increases invasiveness of this non-epithelial tumor [74]. Based on the results presented here, we postulate that the combination of disseminated disease (M1) at diagnosis, which is a well-established clinical prognostic factor in ES, with molecular evaluation of *CDH2* expression may be a better predictor of poor prognosis in ES patients than the metastatic status alone. Such combination clearly distinguished between patients who died of disease and were alive at the last follow-up time point (Fig. 4). Detection of symptoms of systemic disease in ES patients may change the current prognostic scoring model and influence therapeutic decisions. Based on the unfavorable results of hematological and molecular examination of PB specimens, patients with macroscopically localized disease may be qualified for more aggressive treatment, so far dedicated to patients with disseminated disease, such as chemotherapy intensified either by dose escalation, or by the addition of new agents. High-dose chemotherapy with autologous hematopoietic stem cell rescue may also be considered in these patients. Prognostic implications of PB testing in ES patients warrant further studies.

To conclude, our study supports the hypothesis of systemic nature of ES. We show evidence of immune involvement in ES and point to the possible mechanism by which immune system may be involved in osteoclastogenesis intensification and progression in ES. Furthermore, we demonstrate the significant prognostic power of *CDH2* expression in PB cells in terms of OS, especially when combined with metastatic status at the time of routine pathologic diagnosis.

## References

[1] Fletcher CDM, Bridge JA, Hogendoorn P, Mertens F (2013). WHO classification of tumours of soft tissue and bone.

[2] Bernstein M, Kovar H, Paulussen M, Randall RL, Schuck A, Teot LA, Juergens H (2006). Ewing’s sarcoma family of tumors: current management. Oncologist.

[3] Lee JA, Kim DH, Cho J, Lim JS, Koh JS, Yoo JY, Kim MS, Kong CB, Song WS, Cho WH, Lee SY, Jeon DG (2011). Treatment outcome of Korean patients with localized Ewing sarcoma family of tumors: a single institution experience. Jpn J Clin Oncol.

[4] West DC, Grier HE, Swallow MM, Demetri GD, Granowetter L, Sklar J (1997). Detection of circulating tumor cells in patients with Ewing’s sarcoma and peripheral primitive neuroectodermal tumor. J Clin Oncol.

[5] Avigad S, Cohen IJ, Zilberstein J, Liberzon E, Goshen Y, Ash S, Meller I, Kollender Y, Issakov J, Zaizov R, Yaniv I (2004). The predictive potential of molecular detection in the nonmetastatic Ewing family of tumors. Cancer.

[6] Toomey EC, Schiffman JD, Lessnick SL (2010). Recent advances in the molecular pathogenesis of Ewing’s sarcoma. Oncogene.

[7] Ash S, Luria D, Cohen IJ, Goshen Y, Toledano H, Issakov J, Yaniv I, Avigad S (2011). Excellent prognosis in a subset of patients with Ewing sarcoma identified at diagnosis by CD56 using flow cytometry. Clin Cancer Res.

[8] Fujiwara T, Fukushi J, Yamamoto S, Matsumoto Y, Setsu N, Oda Y, Yamada H, Okada S, Watari K, Ono M, Kuwano M, Kamura S, Iida K, Okada Y, Koga M, Iwamoto Y (2011). Macrophage infiltration predicts a poor prognosis for human ewing sarcoma. Am J Pathol.

[9] Taylor R, Knowles HJ, Athanasou NA (2011). Ewing sarcoma cells express RANKL and support osteoclastogenesis. J Pathol..

[10] Desai SS, Jambhekar NA (2010). Pathology of Ewing’s sarcoma/PNET: current opinion and emerging concepts. Indian J Orthop..

[11] Lau YS, Adamopoulos IE, Sabokbar A, Giele H, Gibbons CL, Athanasou NA (2007). Cellular and humoral mechanisms of osteoclast formation in Ewing’s sarcoma. Br J Cancer.

[12] Hanahan D, Weinberg RA (2011). Hallmarks of cancer: the next generation. Cell.

[13] Turc-Carel C, Aurias A, Mugneret F, Lizard S, Sidaner I, Volk C, Thiery JP, Olschwang S, Philip I, Berger MP, Philip T, Lenoir GM, Mazabraud A (1988). Chromosomes in Ewing’s sarcoma. I. An evaluation of 85 cases of remarkable consistency of t(11;22)(q24;q12). Cancer Genet Cytogenet.

[14] Delattre O, Zucman J, Melot T, Garau XS, Zucker JM, Lenoir GM, Ambros PF, Sheer D, Turc-Carel C, Triche TJ, Aurias A, Thomas G (1994). The Ewing family of tumors—a subgroup of small-round-cell tumors defined by specific chimeric transcripts. N Engl J Med.

[15] Zucman J, Melot T, Desmaze C, Ghysdael J, Plougastel B, Peter M, Zucker JM, Triche TJ, Sheer D, Turc-Carel C, Ambros P, Combaret V, Lenoir G, Aurias A, Thomas G, Delattre O (1993). Combinatorial generation of variable fusion proteins in the Ewing family of tumours. EMBO J.

[16] Delattre O, Zucman J, Plougastel B, Desmaze C, Melot T, Peter M, Kovar H, Joubert I, de Jong P, Rouleau G (1992). Gene fusion with an ETS DNA-binding domain caused by chromosome translocation in human tumours. Nature.

[17] May WA, Lessnick SL, Braun BS, Klemsz M, Lewis BC, Lunsford LB, Hromas R, Denny CT (1993). The Ewing’s sarcoma EWS/FLI-1 fusion gene encodes a more potent transcriptional activator and is a more powerful transforming gene than FLI-1. Mol Cell Biol.

[18] Bailly RA, Bosselut R, Zucman J, Cormier F, Delattre O, Roussel M, Thomas G, Ghysdael J (1994). DNA-binding and transcriptional activation properties of the EWS–FLI-1 fusion protein resulting from the t(11;22) translocation in Ewing sarcoma. Mol Cell Biol.

[19] Patócs B, Németh K, Garami M, Arató G, Kovalszky I, Szendrői M, Fekete G (2013). Multiple splice variants of EWSR1-ETS fusion transcripts co-existing in the Ewing sarcoma family of tumors. Cell Oncol Dordr..

[20] Carpentieri DF, Qualman SJ, Bowen J, Krausz T, Marchevsky A, Dickman PS, Cancer Committee, College of American Pathologists (2005). Protocol for the examination of specimens from pediatric and adult patients with osseous and extraosseous ewing sarcoma family of tumors, including peripheral primitive neuroectodermal tumor and ewing sarcoma. Arch Pathol Lab Med.

[21] Bridge RS, Rajaram V, Dehner LP, Pfeifer JD, Perry A (2006). Molecular diagnosis of Ewing sarcoma/primitive neuroectodermal tumor in routinely processed tissue: a comparison of two FISH strategies and RT-PCR in malignant round cell tumors. Mod Pathol.

[22] Mangham DC, Williams A, McMullan DJ, McClure J, Sumathi VP, Grimer RJ, Davies AM (2006). Ewing’s sarcoma of bone: the detection of specific transcripts in a large, consecutive series of formalin-fixed, decalcified, paraffin-embedded tissue samples using the reverse transcriptase-polymerase chain reaction. Histopathology.

[23] Le Deley MC, Delattre O, Schaefer KL, Burchill SA, Koehler G, Hogendoorn PC, Lion T, Poremba C, Marandet J, Ballet S, Pierron G, Brownhill SC, Nesslböck M, Ranft A, Dirksen U, Oberlin O, Lewis IJ, Craft AW, Jürgens H, Kovar H (2010). Impact of EWS–ETS fusion type on disease progression in Ewing’s sarcoma/peripheral primitive neuroectodermal tumor: prospective results from the cooperative Euro-E.W.I.N.G. 99 trial. J Clin Oncol.

[24] van Doorninck JA, Ji L, Schaub B, Shimada H, Wing MR, Krailo MD, Lessnick SL, Marina N, Triche TJ, Sposto R, Womer RB, Lawlor ER (2010). Current treatment protocols have eliminated the prognostic advantage of type 1 fusions in Ewing sarcoma: a report from the Children’s Oncology Group. J Clin Oncol.

[25] Barr FG, Meyer W (2010). Role of fusion subtype in Ewing sarcoma. J Clin Oncol.

[26] Folpe AL, Goldblum JR, Rubin BP, Shehata BM, Liu W, Dei Tos AP, Weiss SW (2005). Morphologic and immunophenotypic diversity in Ewing family tumors: a study of 66 genetically confirmed cases. Am J Surg Pathol.

[27] Daugaard S, Kamby C, Sunde LM, Myhre-Jensen O, Schiødt T (1989). Ewing’s sarcoma. A retrospective study of histological and immunohistochemical factors and their relation to prognosis. Virchows Arch A Pathol Anat Histopathol..

[28] Lizard-Nacol S, Lizard G, Justrabo E, Turc-Carel C (1989). Immunologic characterization of Ewing’s sarcoma using mesenchymal and neural markers. Am J Pathol.

[29] Crowley E, Di Nicolantonio F, Loupakis F, Bardelli A (2013). Liquid biopsy: monitoring cancer-genetics in the blood. Nat Rev Clin Oncol..

[30] Krebs MG, Metcalf RL, Carter L, Brady G, Blackhall FH, Dive C. Molecular analysis of circulating tumour cells-biology and biomarkers. Nat Rev Clin Oncol. 2014; doi:10.1038/nrclinonc.2013.253. 10.1038/nrclinonc.2013.253 24445517

[31] Peter M, Magdelenat H, Michon J, Melot T, Oberlin O, Zucker JM, Thomas G, Delattre O (1995). Sensitive detection of occult Ewing’s cells by the reverse transcriptase-polymerase chain reaction. Br J Cancer.

[32] Pfleiderer C, Zoubek A, Gruber B, Kronberger M, Ambros PF, Lion T, Fink FM, Gadner H, Kovar H (1995). Detection of tumour cells in peripheral blood and bone marrow from Ewing tumour patients by RT-PCR. Int J Cancer.

[33] Toretsky JA, Neckers L, Wexler LH (1995). Detection of (11;22)(q24;q12) translocation-bearing cells in peripheral blood progenitor cells of patients with Ewing’s sarcoma family of tumors. J Natl Cancer Inst.

[34] Fagnou C, Michon J, Peter M, Bernoux A, Oberlin O, Zucker JM, Magdelenat H, Delattre O (1998). Presence of tumor cells in bone marrow but not in blood is associated with adverse prognosis in patients with Ewing’s tumor. Société Française d’Oncologie Pédiatrique. J Clin Oncol.

[35] Schleiermacher G, Peter M, Oberlin O, Philip T, Rubie H, Mechinaud F, Sommelet-Olive D, Landman-Parker J, Bours D, Michon J, Delattre O (2003). Société Française d’Oncologie Pédiatrique. Increased risk of systemic relapses associated with bone marrow micrometastasis and circulating tumor cells in localized Ewing tumor. J Clin Oncol.

[36] DePrimo SE, Wong LM, Khatry DB, Nicholas SL, Manning WC, Smolich BD, O’Farrell AM, Cherrington JM (2003). Expression profiling of blood samples from an SU5416 Phase III metastatic colorectal cancer clinical trial: a novel strategy for biomarker identification. BMC Cancer.

[37] Twine NC, Stover JA, Marshall B, Dukart G, Hidalgo M, Stadler W, Logan T, Dutcher J, Hudes G, Dorner AJ, Slonim DK, Trepicchio WL, Burczynski ME (2003). Disease-associated expression profiles in peripheral blood mononuclear cells from patients with advanced renal cell carcinoma. Cancer Res.

[38] Burczynski ME, Twine NC, Dukart G, Marshall B, Hidalgo M, Stadler WM, Logan T, Dutcher J, Hudes G, Trepicchio WL, Strahs A, Immermann F, Slonim DK, Dorner AJ (2005). Transcriptional profiles in peripheral blood mononuclear cells prognostic of clinical outcomes in patients with advanced renal cell carcinoma. Clin Cancer Res.

[39] Csontos Z, Nádasi E, Csejtey A, Illényi L, Kassai M, Lukács L, Kelemen D, Kvarda A, Zólyomi A, Horváth OP, Ember I (2008). Oncogene and tumor suppressor gene expression changes in the peripheral blood leukocytes of patients with colorectal cancer. Tumori..

[40] Showe MK, Vachani A, Kossenkov AV, Yousef M, Nichols C, Nikonova EV, Chang C, Kucharczuk J, Tran B, Wakeam E, Yie TA, Speicher D, Rom WN, Albelda S, Showe LC (2009). Gene expression profiles in peripheral blood mononuclear cells can distinguish patients with non-small cell lung cancer from patients with nonmalignant lung disease. Cancer Res.

[41] Wolf B, Schwarzer A, Côté AL, Hampton TH, Schwaab T, Huarte E, Tomlinson CR, Gui J, Fisher JL, Fadul CE, Hamilton JW, Ernstoff MS (2012). Gene expression profile of peripheral blood lymphocytes from renal cell carcinoma patients treated with IL-2, interferon-α and dendritic cell vaccine. PLoS ONE.

[42] Palma P, Cuadros M, Conde-Muíño R, Olmedo C, Cano C, Segura-Jiménez I, Blanco A, Bueno P, Ferrón JA, Medina P. Microarray profiling of mononuclear peripheral blood cells identifies novel candidate genes related to chemoradiation response in rectal cancer. PLoS One. 2013;8(9):e74034. doi:10.1371/journal.pone.0074034. eCollection 2013. 10.1371/journal.pone.0074034 PMC3764031 24040155

[43] Ohali A, Avigad S, Zaizov R, Ophir R, Horn-Saban S, Cohen IJ, Meller I, Kollender Y, Issakov J, Yaniv I (2004). Prediction of high risk Ewing’s sarcoma by gene expression profiling. Oncogene.

[44] Cheung IY, Feng Y, Danis K, Shukla N, Meyers P, Ladanyi M, Cheung NK (2007). Novel markers of subclinical disease for Ewing family tumors from gene expression profiling. Clin Cancer Res.

[45] Schaefer KL, Eisenacher M, Braun Y, Brachwitz K, Wai DH, Dirksen U, Lanvers-Kaminsky C, Juergens H, Herrero D, Stegmaier S, Koscielniak E, Eggert A, Nathrath M, Gosheger G, Schneider DT, Bury C, Diallo-Danebrock R, Ottaviano L, Gabbert HE, Poremba C (2008). Microarray analysis of Ewing’s sarcoma family of tumours reveals characteristic gene expression signatures associated with metastasis and resistance to chemotherapy. Eur J Cancer.

[46] Mackintosh C, Ordóñez JL, García-Domínguez DJ, Sevillano V, Llombart-Bosch A, Szuhai K, Scotlandi K, Alberghini M, Sciot R, Sinnaeve F, Hogendoorn PC, Picci P, Knuutila S, Dirksen U, Debiec-Rychter M, Schaefer KL, de Álava E (2012). 1q gain and CDT2 overexpression underlie an aggressive and highly proliferative form of Ewing sarcoma. Oncogene.

[47] Kovar H, Alonso J, Aman P, Aryee DN, Ban J, Burchill SA, Burdach S, De Alava E, Delattre O, Dirksen U, Fourtouna A, Fulda S, Helman LJ, Herrero-Martin D, Hogendoorn PC, Kontny U, Lawlor ER, Lessnick SL, Llombart-Bosch A, Metzler M, Moriggl R, Niedan S, Potratz J, Redini F, Richter GH, Riedmann LT, Rossig C, Schäfer BW, Schwentner R, Scotlandi K, Sorensen PH, Staege MS, Tirode F, Toretsky J, Ventura S, Eggert A, Ladenstein R (2012). The first European interdisciplinary Ewing sarcoma research summit. Front Oncol..

[48] Rutkowski P, Mazurkiewicz T, Krzakowski M, Ptaszyński K, Klepacka T, Grzesiakowska U, Falkowski S, Świtaj T, Nowecki Z, Morysiński T, Spindel J, Chmielik E, Dragan S, Nazar J, Kotrych D, Skowroński J, Szumera-Ciećkiewicz A, Szafrański A, Szumiło J, Karpik M, Balcerkiewicz K, Mazuryk R, Jarosz B, Rychłowska-Pruszyńska M, Rzeszutko M, Nowakowski A, Ryś J, Olszewski WT, Woźniak W, on behalf of Polish Registry of Bone Tumors. Recommendations for diagnostics and therapy of adult patients with malignant primary bone tumors. Onkologia w Praktyce Klinicznej 2010, tom 6, no 6, 355–369.

[49] Vandesompele J, De Preter K, Pattyn F, Poppe B, Van Roy N, De Paepe A, Speleman F. Accurate normalization of real-time quantitative RT-PCR data by geometric averaging of multiple internal control genes. Genome Biol. 2002;3(7):research0034.1–research0034.11. 10.1186/gb-2002-3-7-research0034 PMC126239 12184808

[50] Andersen CL, Jensen JL, Ørntoft TF (2004). Normalization of real-time quantitative reverse transcription-PCR data: a model-based variance estimation approach to identify genes suited for normalization, applied to bladder and colon cancer data sets. Cancer Res.

[51] Livak KJ, Schmittgen TD (2001). Analysis of relative gene expression data using real-time quantitative PCR and the 2(-Delta Delta C(T)) Method. Methods.

[52] World Health Organization. Nutritional Anaemias: Report of a WHO Scientific Group. WHO Technical Reports Series 405. Geneva, Switzerland: World Health Organization, 1968. 4975372

[53] Kang HG, Jenabi JM, Zhang J, Keshelava N, Shimada H, May WA, Ng T, Reynolds CP, Triche TJ, Sorensen PH (2007). E-cadherin cell–cell adhesion in ewing tumor cells mediates suppression of anoikis through activation of the ErbB4 tyrosine kinase. Cancer Res.

[54] Wang H, Kadlecek TA, Au-Yeung BB, Goodfellow HE, Hsu LY, Freedman TS, Weiss A (2010). ZAP-70: an essential kinase in T-cell signaling. Cold Spring Harb Perspect Biol.

[55] Gilmore TD (2006). Introduction to NF-kappaB: players, pathways, perspectives. Oncogene.

[56] Gales D, Clark C, Manne U, Samuel T. The chemokine CXCL8 in carcinogenesis and drug response. ISRN Oncol. 2013;2013:859154. eCollection 2013. 10.1155/2013/859154 PMC3810054 24224100

[57] Savola S, Klami A, Myllykangas S, Manara C, Scotlandi K, Picci P, Knuutila S, Vakkila J (2011). High expression of complement component 5 (C5) at tumor site associates with superior survival in Ewing’s sarcoma family of tumour patients. ISRN Oncol..

[58] Morris JC, Waldmann TA (2000). Advances in interleukin 2 receptor targeted treatment. Ann Rheum Dis.

[59] Kuss I, Saito T, Johnson JT, Whiteside TL (1999). Clinical significance of decreased zeta chain expression in peripheral blood lymphocytes of patients with head and neck cancer. Clin Cancer Res.

[60] Pignataro L, Pagani D, Brando B, Sambataro G, Scarpati B, Corsi MM (2007). Down-regulation of zeta chain and zeta-associated protein 70 (Zap 70) expression in circulating T lymphocytes in laryngeal squamous cell carcinoma. Anal Quant Cytol Histol.

[61] Ciszak L, Kosmaczewska A, Werynska B, Szteblich A, Jankowska R, Frydecka I (2009). Impaired zeta chain expression and IFN-gamma production in peripheral blood T and NK cells of patients with advanced lung cancer. Oncol Rep.

[62] Dworacki G, Meidenbauer N, Kuss I, Hoffmann TK, Gooding W, Lotze M, Whiteside TL (2001). Decreased zeta chain expression and apoptosis in CD3 + peripheral blood T lymphocytes of patients with melanoma. Clin Cancer Res.

[63] Penna D, Müller S, Martinon F, Demotz S, Iwashima M, Valitutti S (1999). Degradation of ZAP-70 following antigenic stimulation in human T lymphocytes: role of calpain proteolytic pathway. J Immunol..

[64] Zhang H, Merchant MS, Chua KS, Khanna C, Helman LJ, Telford B, Ward Y, Summers J, Toretsky J, Thomas EK, June CH, Mackall CL (2003). Tumor expression of 4-1BB ligand sustains tumor lytic T cells. Cancer Biol Ther.

[65] Hruz T, Laule O, Szabo G, Wessendorp F, Bleuler S, Oertle L, Widmayer P, Gruissem W, Zimmermann P. Genevestigator V3: a reference expression database for the meta-analysis of transcriptomes. Adv Bioinform. 2008;2008, Article ID 420747. doi:10.1155/2008/420747. 10.1155/2008/420747 PMC2777001 19956698

[66] Kong YY, Feige U, Sarosi I, Bolon B, Tafuri A, Morony S, Capparelli C, Li J, Elliott R, McCabe S, Wong T, Campagnuolo G, Moran E, Bogoch ER, Van G, Nguyen LT, Ohashi PS, Lacey DL, Fish E, Boyle WJ, Penninger JM (1999). Activated T cells regulate bone loss and joint destruction in adjuvant arthritis through osteoprotegerin ligand. Nature.

[67] Colucci S, Brunetti G, Rizzi R, Zonno A, Mori G, Colaianni G, Del Prete D, Faccio R, Liso A, Capalbo S, Liso V, Zallone A, Grano M (2004). T cells support osteoclastogenesis in an in vitro model derived from human multiple myeloma bone disease: the role of the OPG/TRAIL interaction. Blood.

[68] Lari R, Kitchener PD, Hamilton JA (2009). The proliferative human monocyte subpopulation contains osteoclast precursors. Arthritis Res Ther..

[69] Sica A, Allavena P, Mantovani A (2008). Cancer related inflammation: the macrophage connection. Cancer Lett.

[70] Mbalaviele G, Chen H, Boyce BF, Mundy GR, Yoneda T (1995). The role of cadherin in the generation of multinucleated osteoclasts from mononuclear precursors in murine marrow. J Clin Invest..

[71] Shin CS, Her SJ, Kim JA, Kim DH, Kim SW, Kim SY, Kim HS, Park KH, Kim JG, Kitazawa R, Cheng SL, Civitelli R (2005). Dominant negative N-cadherin inhibits osteoclast differentiation by interfering with beta-catenin regulation of RANKL, independent of cell–cell adhesion. J Bone Miner Res.

[72] Crouser ED, Shao G, Julian MW, Macre JE, Shadel GS, Tridandapani S, Huang Q, Wewers MD (2009). Monocyte activation by necrotic cells is promoted by mitochondrial proteins and formyl peptide receptors. Crit Care Med.

[73] Ruka W, Rutkowski P, Kaminska J, Rysinska A, Steffen J (2001). Alterations of routine blood tests in adult patients with soft tissue sarcomas: relationships to cytokine serum levels and prognostic significance. Ann Oncol.

[74] Péglion F, Etienne-Manneville S (2012). N-cadherin expression level as a critical indicator of invasion in non-epithelial tumors. Cell Adh Migr..

